# Construction and validation of risk prediction model for gastrointestinal bleeding in patients after coronary artery bypass grafting

**DOI:** 10.1038/s41598-023-49405-6

**Published:** 2023-12-11

**Authors:** Mei Yang, Shuyu Zhan, Han Gao, Caiyun Liao, Shisi Li

**Affiliations:** Department of Cardiovascular Surgery, The Second Affiliated Hospital of Army Medical University, Chongqing, China

**Keywords:** Cardiology, Gastroenterology

## Abstract

This study aimed to develop a risk prediction model for gastrointestinal bleeding in patients after coronary artery bypass grafting (CABG) and assessed its accuracy. A retrospective analysis was conducted on 232 patients who underwent CABG under general anesthesia in our hospital between January 2022 and December 2022. The patients were divided into gastrointestinal bleeding (GIB) group (n = 52) and group without gastrointestinal bleeding (non-GIB) (n = 180). The independent risk factors for gastrointestinal bleeding in post-CABG patients were analyzed using χ^2^ test, t test and logistic multivariate regression analysis. A prediction model was established based on the identified risk factors. To verify the accuracy of the prediction model, a verification group of 161 patients who met the criteria was selected between January to June 2023, and the Bootstrap method was used for internal validation. The discrimination of the prediction model was evaluated using the area under the curve (AUC), where a higher AUC indicates a stronger discrimination effect of the model. The study developed a risk prediction model for gastrointestinal bleeding after CABG surgery. The model identified four independent risk factors: duration of stay in the intensive care unit (ICU) (OR 0.761), cardiopulmonary bypass time (OR 1.019), prolonged aortic occlusion time (OR 0.981) and re-operation for bleeding (OR 0.180). Based on these factors, an individualized risk prediction model was constructed. The C-index values of the modeling group and the verification group were 0.805 [95% CI (0.7303–0.8793)] and 0.785 [95% CI (0.6932–0.8766)], respectively, which indicated a good accuracy and discrimination of this model. The calibration and standard curves showed similar results, which further supported the accuracy of the risk prediction model. In conclusion, ICU time, cardiopulmonary bypass time, aortic occlusion time and re-operation for bleeding are identified as independent risk factors for gastrointestinal bleeding in patients after CABG. The risk prediction model developed in this study demonstrates strong predictive performance and provides valuable insights for clinical medical professionals in evaluating gastrointestinal complications in CABG patients.

## Introduction

Coronary atherosclerotic heart disease (CHD) is a prevalent cardiovascular disease. Coronary artery bypass grafting (CABG) is recognized as the most effective treatment method for this disease^1^. According to the ‘2022 China Cardiovascular Health and Disease Report’^2^, as the prevalence and mortality of cardiovascular diseases increase in our country, the number of patients undergoing CABG surgery is also rising. Advancements in science, technology, surgical techniques and perioperative care have expanded the selection criteria for cardiac surgery to include high-risk patients. However, this might lead to an increase in the probability of complications and the complexity of the surgery^3^. Studies have indicated a positive correlation between gastrointestinal bleeding (GIB) after cardiac surgery and rapid postoperative recovery as well as mortality^4,5^. The use of multiple drugs post-surgery can mask the patient’s vital signs and symptoms, making early identification of postoperative gastrointestinal bleeding challenging. Therefore, the prevention, early diagnosis and treatment of gastrointestinal bleeding after CABG are of great importance in clinical practice.

This study investigated the risk factors associated with gastrointestinal bleeding in patients after CABG and developed a risk prediction model to aid medical professionals in identifying the potential risk of gastrointestinal bleeding early on. The study aimed to enhance the understanding of gastrointestinal bleeding and provided predictive care for high-risk patients. Reducing the incidence of perioperative gastrointestinal bleeding in CABG patients is crucial for improving patient prognosis and facilitating speedy recovery of the heart.

## Patients and methods

### Study population

According to the inclusion and exclusion criteria, we selected data from 232 patients who underwent CABG in our hospital from January 2022 to December 2022 (Table 1) for constructing a risk prediction model. Antiplatelet drugs, including clopidogrel and ticagrelor, were discontinued for 7 days and 5 days in patients before surgery, respectively. The data mainly included patient demographics, preoperative risk factors, intraoperative and postoperative data, and so on. To verify the prediction effect of the model, a verification group of 161 patients from January to June 2023 was enrolled. The inclusion criteria for patients were: (1) Age ≥ 18 years old; (2) Undergoing CABG alone. The exclusion criteria were: (1) Patients undergoing CABG combined with valve replacement; (2) Patients with gastrointestinal bleeding before surgery. Gastrointestinal bleeding was defined as vomiting of coffee-like material and passing large amounts of tarry or bloody stools^6^. This study is a retrospective study and has been approved by the Ethics Committee of the Second Affiliated Hospital of Army Medical University with exempting patients from informed consent. The current study was designed in accordance with TRIPOD guidelines.

**Table 1:** Univariate analysis of patient demographic data, preoperative risk factors and surgical data.

	GIB (n = 52)	non-GIB (n = 180)	P	t/χ^2^
Age (years)	63.87 ± 8.49	60.20 ± 9.44	0.012	2.52
Age ≥ 65 years	26 (50.0)	67 (37.2)	0.032	4.61
Sex (Male)	46 (88.5)	145 (80.6)	0.188	1.73
EF (%)	60.87 ± 9.20	54.02 ± 14.08	< 0.001	4.15
NYHA ≥ 3	39 (75.0)	120 (66.7)	0.254	1.30
Cerebral infarction	9 (17.3)	18 (10.0)	0.148	2.10
Hypertension	34 (65.4)	105 (58.3)	0.361	0.84
Diabetes	21 (40.4)	67 (37.2)	0.679	0.17
Digestive tract diseases	8 (15.4)	20 (11.1)	0.405	0.69
Smoke	39 (75.0)	114 (63.3)	0.118	2.45
MI	12 (23.1)	43 (23.9)	0.903	0.02
Number of diseased vessels	3.37 ± 0.74	3.12 ± 0.90	0.075	1.79
PPI use	8 (15.4)	32 (17.8)	0.687	0.16
Aggrastat use	27 (51.9)	123 (68.3)	0.029	4.75
LMWHC use	18 (34.6)	33 (18.3)	0.013	6.24
Blood platelet	198.06 ± 60.98	198.83 ± 62.76	0.938	0.08
INR	1.02 ± 0.10	1.02 ± 0.07	0.885	0.15
ALT	32.09 ± 44.59	34.80 ± 27.14	0.591	0.54
AST	27.65 ± 30.63	28.13 ± 34.01	0.927	0.09
GGT	39.42 ± 33.97	40.63 ± 33.89	0.821	0.23
Total bilirubin	17.98 ± 6.52	15.27 ± 10.32	0.392	0.86
Albumin	41.47 ± 5.55	44.16 ± 4.40	< 0.001	3.65
Prealbumin	245.23 ± 59.72	255.57 ± 57.69	0.260	1.13
Urea	7.37 ± 2.84	6.09 ± 2.31	< 0.001	3.33
Creatinine	95.60 ± 38.97	83.19 ± 26.69	0.009	2.64
GFR	76.33 ± 21.26	84.77 ± 18.88	0.006	2.76
Uric Acid	285.68 ± 110.40	361.82 ± 97.96	0.134	1.51
CPB use	38 (73.1)	78 (43.3)	< 0.001	14.28
CPB time	121.79 ± 90.52	67.96 ± 95.19	< 0.001	3.63
ACCT	64.00 ± 54.84	31.59 ± 56.64	< 0.001	3.66
Intraoperative transfusion	38 (73.1)	83 (46.1)	< 0.001	11.76
Time of operation	382.67 ± 93.27	339.29 ± 100.15	0.006	2.79
Mechanical ventilation time	7660.52 ± 8456.67	2377.25 ± 4780.31	< 0.001	5.79
ICU stay time	8.79 ± 8.10	3.3 ± 1.75	< 0.001	8.42
IABP use	16 (30.8)	31 (17.2)	0.032	4.58
Re-operation for bleeding	11 (21.2)	6 (3.3)	< 0.001	18.87
Arrhythmia	28 (53.8)	35 (19.4)	< 0.001	24.14
Low cardiac output syndrome	16 (30.8)	32 (17.8)	0.042	4.15
Infection	30 (57.7)	39 (21.7)	< 0.001	25.06

The results were expressed as mean ± standard deviation or n (%).

*EF* left ventricular ejection fraction, *MI* myocardial infarction, *PPI* proton pump preparation, *INR* international normalized ratio, *ALT* alanine aminotransferase, *AST* aspartate aminotransferase, *GGT* gamma-glutamyltransferase, *GFR* glomerular filtration rate, *CPB* cardiopulmonary bypass, *ACCT* aorticcross-clamptime, *ICU* intensive care unit, *IABP* intra-aortic ballon pump, *LMWHC* low molecular-weight heparin calcium.

### Variable selection

Based on a review of domestic and foreign literature and consultation with senior medical staff in the Department of Gastroenterology, potential risk factors were identified. These selected risk factors with a strong theoretical basis, clinical significance and an information collection questionnaire were developed. The study involved retrieving electronic medical records of patients and collecting demographic information, disease diagnosis and treatment details of the research subjects. A comparison was made between patients who experienced gastrointestinal bleeding after heart surgery and those who underwent surgery during the same study period but did not experience bleeding. Additional measurement indicators in the study included postoperative complications such as re-operation for bleeding, arrhythmia, low cardiac output syndrome, use of Intra-Aortic Balloon Pump (IABP) and infection. Arrhythmia was defined as abnormal findings on electrocardiogram (ECG) indicating extra beats, supraventricular tachycardia, ventricular arrhythmias or bradyarrhythmias. Low cardiac output syndrome is characterized by a cardiac index lower than 2.2 L/(min∙m^2^) along with peripheral vasoconstriction and tissue hypoperfusion, which was measured by placing a pulmonary artery (PA) catheter in all patients of the study. All patients enrolled in the study underwent thromboelastography examination before surgery. Following the latest evidence-based guidelines, as outlined in the 2022 version of the Chinese expert consensus on secondary prevention after CABG^2^, patients who underwent CABG surgery in the study were prescribed long-term oral antiplatelet therapy, primarily consisting of aspirin (100 mg qd). In our current study, proton pump inhibitor (PPI) preparations were initiated within 24 h after surgery to prevent gastrointestinal bleeding, and enteral nutrition was introduced as early as possible after the patient regained consciousness from anesthesia to promote intestinal motility restoration. Test results were collected based on the most recent preoperative reference.

### Data collection

Prior to commencing the study, the medical personnel responsible for data collection underwent training and were informed about the main research objective and methodology. Patients were screened based on specific inclusion and exclusion criteria. To ensure accurate documentation of the research subjects’ actual circumstances, a standardized questionnaire was employed for data entry. Additionally, a designated individual regularly conducted sampling inspections and logical verifications to validate the accuracy of the collected data. A random selection of data collected was verified and any erroneous data was corrected weekly.

The inclusion and exclusion criteria should be strictly understood to ensure that all CABG patients who have been continuously observed for a specific period of time were selected for the dataset. This could ensure an adequate sample size and comparability in baseline characteristics between the two analyzed groups of data. To minimize the impact of confounding factors on the experimental results, randomization should be strictly implemented during the experiment. Additionally, the researchers refrained from participating in the data collection and entry process to control bias and prevent subjectivity.

### Intraoperative and postoperative management

All patients included in the study underwent CABG under general anesthesia, and sternotomy was performed on all patients. For patients who underwent extracorporeal circulation, two vena cava were used between the ascending aorta and the right atrium to initiate extracorporeal circulation after systemic heparinization. Protamine was then administered to neutralize the heparin. After the completion of the operation, all patients were transferred to the intensive care unit. Once they were hemodynamically stable and fully conscious, patients were gradually weaned off the ventilator. Stable patients were either transferred to general wards or discharged home.

### Statistical analyses

SPSS 27.0 was used to analyze the clinical data of the patients. The qualitative data were analyzed using χ^2^ test. T-test was used for the quantitative data with a normal distribution. A significance level of P < 0.05 was considered statistically significant. The risk factors were determined through univariate and logistic multivariate regression analysis. The nomogram prediction model was developed using R 4.2.3 and the rms equation package. The model was internally validated using the caret software package and the repeated sampling method (Bootstrap method), and externally validated using a separate verification set. The calibration curve and C-index were used to assess the predictive performance of the nomogram.

### Ethical approval

The studies involving human participants were reviewed and approved by Xinqiao Hospital (the Second Affiliated Hospital of Army Medical University) Academic Ethics Committee.

## Results

A total of 232 adult patients were enrolled in this study. The mean age was 61 ± 9.3 years. Eleven patients (4.7%) were older than 75 years, and 191 patients (82%) were male. Preoperative risk factors included hypertension (n = 139, 60%), diabetes (n = 88, 38%), cerebral infarction (n = 27, 12%), myocardial infarction (n = 24, 24%), gastrointestinal disease (n = 28, 12%), and smoking (n = 153, 66%). Proton pump preparations were used prophylactically in 47 patients (20%). The average ejection fraction of the included patients was 59.4% ± 10.6%. Eleven percent (n = 25) of these patients had a left ventricular ejection fraction of less than 45%. The average duration of CABG under general anesthesia was 349 ± 100.1 min, and 116 patients (50%) underwent cardiopulmonary bypass. Intraoperative blood transfusion was received by 121 patients (52%). The average duration of intensive care unit (ICU) stay was 4.6 ± 4.7 days, and the average duration of mechanical ventilation was 3561 ± 6194.5 min. Aortic balloon pump was used to treat 45 patients (19%).

Gastrointestinal bleeding occurred in 52 (22%) patients following the operation. Among these patients, 11 (21%) required postoperative re-operation for bleeding, while 28 (54%) experienced postoperative arrhythmia. Additionally, 19 (37%) patients developed postoperative low cardiac output syndrome and 30 (58%) patients were diagnosed as postoperative infection. Unfortunately, 12 patients, accounting for 23% of the group with gastrointestinal bleeding, were registered as deaths.

### Univariate analysis of gastrointestinal bleeding in patients after CABG

The clinical data of patients who experienced gastrointestinal bleeding after CABG were analyzed using univariate analysis compared with non-GIB group patients. The factors that showed some difference before the operation in patients with gastrointestinal bleeding included age, ejection fraction (EF) ≤ 45%, anticoagulant use prior to the operation, albumin level, urea level, creatinine level, and glomerular filtration rate. Some different factors during the operation included duration of the operation, intraoperative blood transfusion therapy, use of extracorporeal circulation, ICU stay of 3 days or longer, mechanical ventilation time of 3 days or longer and IABP treatment were characterized. Moreover, re-operation for bleeding, postoperative arrhythmia, low cardiac output syndrome and lung infection after surgery showed differently in two groups. All of these factors with a p-value less than 0.05 were presented in the Table 1.

### Logistic regression analysis of gastrointestinal bleeding in patients after CABG

In the logistic multivariate regression analysis, whether the patients complicated gastrointestinal bleeding was identified as the dependent variable. The different variables in the univariate analysis were analyzed. The results of the logistic multivariate analysis revealed that ICU time (OR 0.761, P = 0.008), extracorporeal circulation time (OR 1.019, P = 0.025), aortic occlusion time (OR 0.981, P = 0.047), and re-operation for bleeding (OR 0.180, P = 0.026) were identified as independent predictors of gastrointestinal bleeding after CABG (Table 2).

**Table 2:** Logistic regression analysis of gastrointestinal bleeding in patients after CABG.

	Coef	Standard error	Wald x^2^	P	OR	95% CI	
Constant	− 0.595	6.351	0.009	0.925	0.551	–	
CPB time	− 0.019	0.008	4.996	0.025	0.981	0.965	0.998
ACCT	0.019	0.009	3.94	0.047	1.019	1	1.038
ICU stay time	0.273	0.103	7.033	0.008	1.315	1.074	1.609
Re-operation for bleeding	1.715	0.769	4.974	0.026	5.556	1.231	25.076

### Construction and verification of a nomogram model for predicting gastrointestinal bleeding in patients after CABG

**Figure 1:**
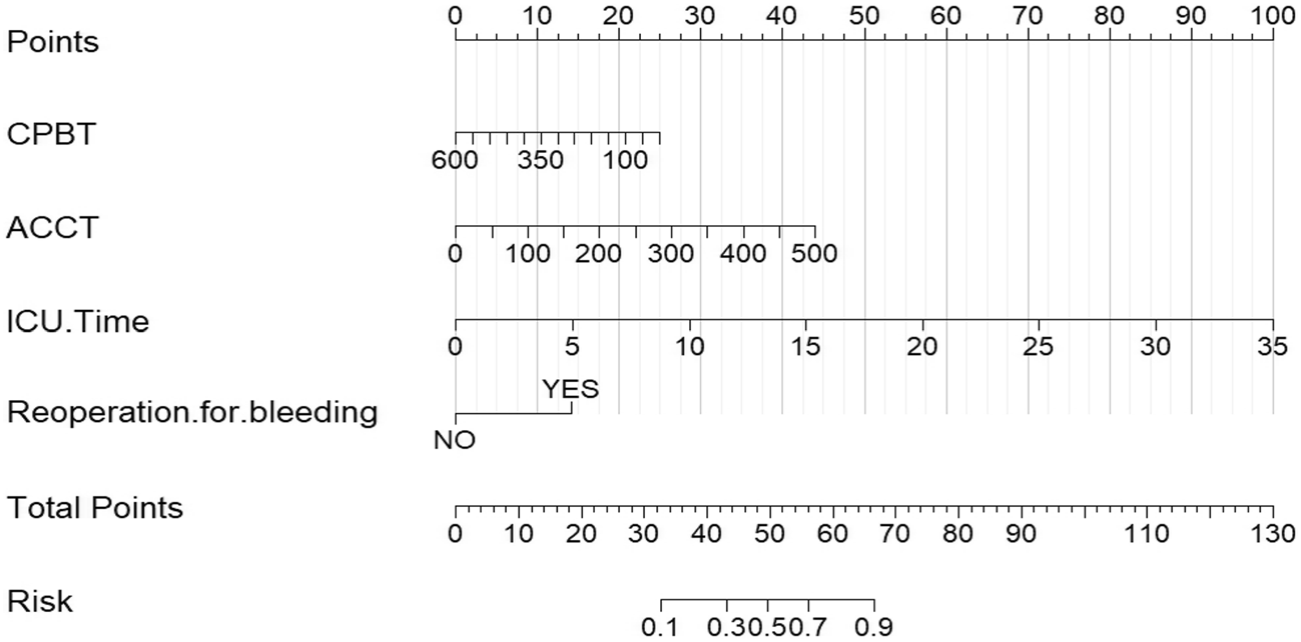
Nomogram model for predicting gastrointestinal bleeding in patients after CABG.

In this study, a nomogram model was further constructed to predict the occurrence of gastrointestinal bleeding in patients after CABG, based on the four independent risk factors that were screened. The model was illustrated in Fig. 1.

**Figure 2:**
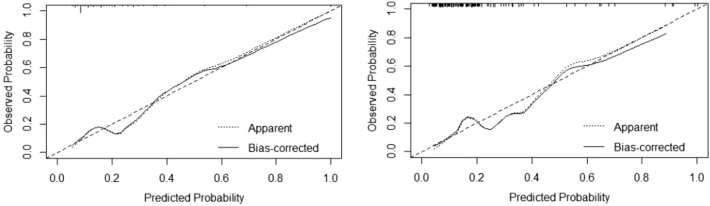
Calibration curves for validation of nomogram predictions (modeling group on the left, validation group on the right).

The model’s internal verification was conducted by repeatedly sampling the original data from the modeling set 1000 times. The external verification was performed on the validation group. The C-index values for the modeling group and the validation group were 0.805 [95% CI (0.7303–0.8793)] and 0.785 [95% CI (0.6932–0.8766)], respectively. The calibration curve verification demonstrated a good fit for both the validation group and the modeling group (Fig. 2).

**Figure 3:**
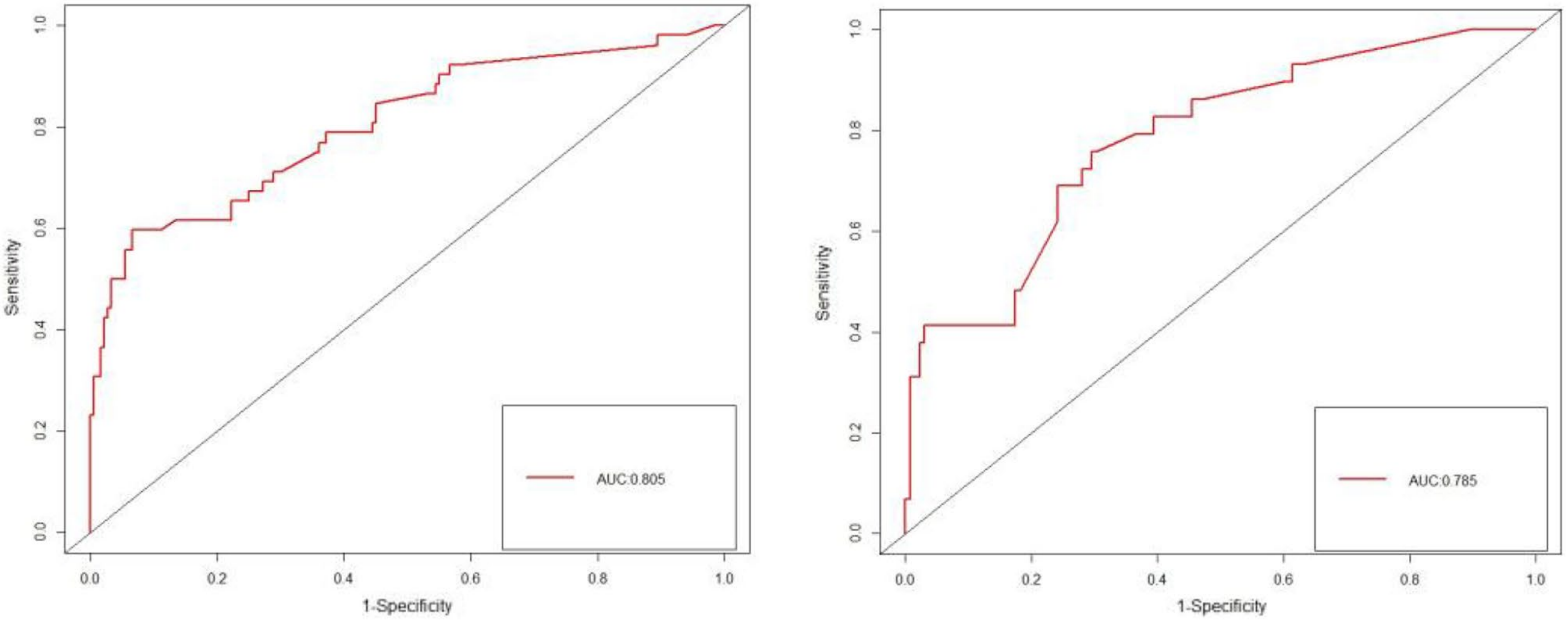
Histogram was verified by ROC curve (Modeling group on the left, validation group on the right).

The ROC curve demonstrated that the AUC for the modeling group and verification group were 0.805 and 0.785, respectively (Fig. 3). These results indicated that the nomogram prediction model exhibited a high level of precision in its predictions.

## Discussion

### Status and hazards of gastrointestinal bleeding in patients with postoperative CABG

A total of 232 patients who underwent CABG were included in this study. Out of these, 52 patients (22.41%) experienced gastrointestinal bleeding, which was higher compared to previous studies^7–9^. It is important to note that stress ulcers and peptic ulcers could contribute to the occurrence of gastrointestinal bleeding^10^. To prevent the occurrence of stress ulcers, nurses should focus on assessing the patient’s past medical history and gastrointestinal function. Gastrointestinal bleeding can increase the mortality rate of patients and the incidence of serious complications such as acute cholecystitis, pancreatitis and peptic ulcers. Studies^11^ have demonstrated that early oral feeding could reduce gastrointestinal complications, promote faster recovery of gastrointestinal function and shorten hospital stays. In this study, no cases of related complications were reported, which may be attributed to the medical staff’s attention to gastrointestinal bleeding during the perioperative period and the implementation of high-quality nursing measures. The evaluation of patients’ gastrointestinal function and provision of healthy diet education during the perioperative period may have improved patient outcomes. However, it is important to note that the small sample size and single-center nature of the study may have influenced these results.

Our findings on the influencing factors of gastrointestinal bleeding in patients after CABG were similar with previous reports. The results of this study indicated that prolonged intraoperative cardiopulmonary bypass time (> 120 min) and aortic occlusion time (> 90 min) were independent risk factors for gastrointestinal bleeding in patients after CABG, which aligned with findings in some other studies^4,4,12^. Surgical trauma, intraoperative hypothermia and non-pulsatile perfusion resulting from CABG under cardiopulmonary bypass were significant factors contributing to the occurrence of adverse complications in the body^13^. Therefore, minimizing the duration of cardiopulmonary bypass and aortic occlusion during the operation could help protect the myocardium, minimize the adverse effects of cardiopulmonary bypass and reduce the incidence of perioperative complications. Additionally, the study found that a postoperative ICU stay exceeding 3 days was an independent risk factor for GIB after CABG. Prolonged ICU stay increased the risk of gastrointestinal bleeding, possibly due to the patients’ inability to eat or resume normal gastrointestinal motility during this period resulting in gastrointestinal complications and subsequent bleeding. Furthermore, the study highlighted that re-operation for bleeding was also an independent risk factor for gastrointestinal bleeding in patients after CABG. Re-operation for bleeding is often associated with low cardiac output and systemic hypotension^14^, which can lead to inadequate blood supply to the gastrointestinal tract, resulting in gastrointestinal complications and eventual bleeding.

### Importance of the risk prediction model for gastrointestinal bleeding in patients after CABG

We further constructed a model for predicting the occurrence of gastrointestinal bleeding in patients after CABG, and evaluate the accuracy of our verification model in this context. The risk prediction model developed in this study was both scientifically valid and feasible. The model’s accuracy was assessed by using the ROC curve and the Bootstrap method for internal validation. The results demonstrated that the predicted outcomes of the model aligned well with the actual incidence rate. According to the ROC curve, an area under the curve (AUC) of 0.500–0.700 indicated low diagnostic value, while an AUC of 0.700–0.900 suggesting a medium prediction effect that was considered acceptable. An AUC exceeding 0.900 indicates a good prediction effect^15^. In this study, the C-index values for the modeling group and the verification group were 0.805 [95% CI (0.730–0.879)] and 0.785 [95% CI (0.693–0.877)], respectively, with corresponding AUCs of 0.805 and 0.785. These results indicated a good predictive power. By utilizing this model, nurses cloud effectively evaluate and identify the risk factors associated with gastrointestinal bleeding after CABG, enhance their understanding of this complication and provide predictive care for high-risk patients to reduce postoperative complications. This model also served as a valuable reference and foundation for developing nursing intervention plans targeting gastrointestinal bleeding following CABG.

## Conclusion

In the current study, ICU time, cardiopulmonary bypass time, aortic occlusion time and re-operation for bleeding were identified as independent risk factors for gastrointestinal bleeding in patients after CABG. Additionally, a risk prediction model for gastrointestinal bleeding after CABG was developed and validated. The results demonstrated that the model exhibited good predictability and could effectively identify high-risk patients with postoperative gastrointestinal bleeding. This information could serve as a reference for determining the need for predictive care. It should be noting that this study was a single-center retrospective study with a small sample size, which might exist some bias. Future multi-center research should be conducted to validate and enhance the prediction model, thereby providing more robust clinical guidance.

## Data Availability

The original contributions presented in the study are included in the article, further inquiries can be directed to the corresponding author.
